# Photoperiod-Dependent Effects of 4-*tert*-Octylphenol on Adherens and Gap Junction Proteins in Bank Vole Seminiferous Tubules

**DOI:** 10.1155/2013/134589

**Published:** 2013-05-12

**Authors:** Anna Hejmej, Malgorzata Kotula-Balak, Katarzyna Chojnacka, Paulina Kuras, Marta Lydka-Zarzycka, Barbara Bilinska

**Affiliations:** Department of Endocrinology, Institute of Zoology, Jagiellonian University, Gronostajowa 9, 30-387 Krakow, Poland

## Abstract

In the present study we evaluated *in vivo* and *in vitro* effects of 4-*tert*-octylphenol (OP) on the expression and distribution of adherens and gap junction proteins, N-cadherin, **β**-catenin, and connexin 43 (Cx43), in testes of seasonally breeding rodents, bank voles. We found that in bank vole testes expression and distribution of N-cadherin, **β**-catenin, and Cx43 were photoperiod dependent. Long-term treatment with OP (200 mg/kg b.w.) resulted in the reduction of junction proteins expressions (*P* < 0.05, *P* < 0.01) and their delocalization in the testes of males kept in long photoperiod, whereas in short-day animals slight increase of Cx43 (*P* < 0.05), N-cadherin, and **β**-catenin (statistically nonsignificant) levels was observed. Effects of OP appeared to be independent of FSH and were maintained during *in vitro* organ culture, indicating that OP acts directly on adherens and gap junction proteins in the testes. An experiment performed using an antiestrogen ICI 182,780 demonstrated that the biological effects of OP on **β**-catenin and Cx43 involve an estrogen receptor-mediated response. Taken together, in bank vole organization of adherens and gap junctions and their susceptibility to OP are related to the length of photoperiod. Alterations in cadherin/catenin and Cx43-based junction may partially result from activation of estrogen receptor **α** and/or **β** signaling pathway.

## 1. Introduction

 Alkylphenols (e.g., 4*-tert*-octylphenol, OP; 4-*tert*-nonylphenol, NP) and alkylphenol ethoxylates are a group of endocrine-disrupting chemicals that accumulate at high concentrations in air, soil and aquatic environment from the use of detergents, paints, pesticides, and plastic manufacturing [1]. They are also found in the fluids and body fat of animals and humans [2]. These compounds are classified as xenoestrogens, since they are able to induce inappropriate estrogenic action or interfere with the actions of endogenous estrogens affecting reproductive development and functions of laboratory and wild animals, and most likely humans [3]. Alkylphenols bind to the nuclear estrogen receptors (ERs), but their binding affinity and ability to activate ER-mediated functions is 100–10,000 times less potent than that of 17*β*-estradiol [4]. However, they exhibit quite robust nongenomic activity [5]. Since 2000, European Union restricted use of alkylphenols as priority hazardous substances by Directive 2000/60/EC [6]. Nevertheless, they are still detected world widely in the environment and organisms [7]. Recent analyses indicate that although alkylphenol ethoxylates levels have been decreasing in the last years, Europe is much more contaminated than North America and developing countries [1].

 Spermatogenesis, a process by which spermatogonia undergo a series of divisions and differentiation to become spermatozoa, is tightly controlled by Sertoli cells. Direct interactions between Sertoli cells and between Sertoli and germ cells are mediated by proteins that form specialized cell junctions. N-cadherin and *β*-catenin are constituent proteins of testicular adherens junctions. They form complexes localized between Sertoli cells at the site of blood-testis barrier (BTB) and between Sertoli cells and elongated spermatids, in the apical ectoplasmic specializations. Cadherin/catenin complexes play a determining role in stabilizing cell-cell contacts and their restructuring during movement of preleptotene spermatocytes across the BTB. It was reported that the altered expression or loss of the protein-protein interactions between N-cadherin and *β*-catenin induces germ cell detachment from the seminiferous epithelium [8, 9]. Connexin 43 (Cx43), the predominant gap junction protein in seminiferous epithelium, is of absolute requirement for normal testicular development and spermatogenesis [10]. In adult testis Cx43 is a component of the junctional complex enabling direct communication and exchange of small molecules between adjacent Sertoli cells, Sertoli and germ cells, and between Leydig cells. Recent studies provided clear evidence that Cx43 can also control spermatogenesis through regulation of tight and anchoring junctions that are closely intermingled with each other at the site of the BTB [11].

 Recent papers have reported that xenoestrogens can affect spermatogenesis by perturbing direct cell-cell interactions in the seminiferous epithelium. Extensively studied xenoestrogen bisphenol A was demonstrated to cause a dose-dependent reduction in several junction proteins (including N-cadherin, *β*-catenin, and Cx43) in Sertoli cells *in vitro *and to disrupt the BTB when administered to immature rats [12, 13]. The estrogenic actions of alkylphenols on the testis are less studied when compared with bisphenol A. Although it was demonstrated that exposure to OP or NP induces changes in multiple gene transcription, cell proliferation, and hormone production *in vitro* and *in vivo*, scarce data are available on their influence on junction proteins in the testes [14–16].

 In our previous study bank voles, seasonally breeding rodents, were used to investigate the effects of OP on male reproductive organs, depending on the length of exposure and reproductive status of animals [17]. We found that long-term exposure of adult males affected expressions of 3*β*-hydroxysteroid dehydrogenase, aromatase, estrogen receptor *α*, and androgen receptor as well as sex steroids levels in the testes and seminal vesicles. Concomitantly, increased apoptosis and, occasionally, germ cell sloughing were found. Interestingly, a subtle difference in the sensitivity to OP between voles kept in different light conditions was noted [17]. To better understand the mechanism of OP-induced alterations in the testis, the present study was aimed to examine changes in the distribution and expression of cell junction proteins, N-cadherin, *β*-catenin, and connexin 43, in bank vole following OP exposure *in vivo*. Importantly, to our knowledge this is the first *in vivo* study on the effect of OP on adherens and gap junction proteins in the testes. In addition, to evaluate potential direct effects of OP on junction protein expressions in seminiferous epithelium and to examine whether these effects are mediated through binding OP to ERs, organ culture model was used. 

## 2. Material and Methods

### 2.1. Animals

 Bank voles (*Clethrionomys glareolus*, Schreber) were obtained from our own colony (Department of Endocrinology, Institute of Zoology, Jagiellonian University, Krakow, Poland) which have been reared under long light cycles (18 h light and 6 h darkness; 18L : 6D) or short light cycles (6 h light and 18 h darkness; 6L : 18D). The animal rooms were maintained at a temperature of 18°C and a relative humidity of 55 ± 5%. Voles were housed in polyethylene cages (42 × 27 × 18 cm^3^) furnished with sawdust and wood shavings for bedding. A standard pelleted diet (LSM diet, Agropol, Motycz, Poland; total isoflavone content below 450 mg/kg diet) supplemented with seeds of wheat or oat, red beet, apples, and water was provided *ad libitum.*

### 2.2. Exposure *In Vivo*

 Twenty-four mature male bank voles (60–70-day-old) kept in long light cycles (18L : 6D, *n* = 12) and short light cycles (6L : 18D, *n* = 12) were dosed orally with 4-*tert*-octylphenol (OP, 200 mg/kg body weight; Sigma-Aldrich, St Louis, MO, USA) every Monday, Wednesday, and Friday for 30 (OP30) or 60 days (OP60). A total of four experimental groups were formed (*n* = 6/each group). 4-*tert*-octylphenol was dissolved in a minimum amount of absolute ethanol and then a measured amount of sesame oil (Sigma-Aldrich, St Louis, MO, USA) was added in 1 : 14 (v/v), as previously described in detail [17]. A dose of exposure to OP was based on the literature [15, 18] and it was finally selected upon our preliminary study in which lower doses (50 and 100 mg/kg body weight) had no effects on bank vole reproductive organs weight and morphology.

 The control animals (*n* = 24) for each experimental group (*n* = 6/each group) were given a vehicle only (ethanol + sesame oil). After the animals were sacrificed by cervical dislocation, testes were immediately excised and serum was collected and frozen in −20°C.

### 2.3. Ethics of Experimentation

 Experiments were performed in accordance with Polish legal requirements, under the license given by the Local Ethics Committee at the Jagiellonian University, Krakow, Poland (No. 88/IV/2010).

### 2.4. Tissue Preparation

 For immunohistochemical analysis one testis from each animal was immersed immediately in 4% formaldehyde and embedded in paraplast (Sigma-Aldrich, St Louis, MO, USA). Sections of 5 *μ*m in thickness were mounted on slides coated with 3-aminopropyltriethoxysilane (Sigma-Aldrich, St Louis, MO, USA), deparaffinized, and rehydrated through decreasing alcoholic solutions. For Western blot and ELISA analysis, contralateral testis from each animal was frozen and stored in −80°C. 

### 2.5. Organ Culture and Exposure *In Vitro*

 Twenty-four untreated male bank voles (60–70-day-old) reared under long light cycles or short light cycles were sacrificed and testes were immediately removed and trimmed free of excess fat and connective tissue. After the capsule had been removed, the testes were cut into small pieces of 2-3 mm in diameter and were placed on Millipore filters (pore size 0.4 *μ*m) (Millipore Corporation, Billerica, MA, USA). The filters were floated on 1.5 mL culture Waymouth's media (Gibco, Grand Island, NY, USA) supplemented with 5% fetal bovine serum (Sigma-Aldrich, St Louis, MO, USA) and L-glutamine containing 50 U/mL penicillin, 50 *μ*g/mL streptomycin in tissue culture dishes and incubated at 37°C, in a humidified atmosphere containing 95% air: 5% CO_2_, for 24 h. 4-*tert*-octylphenol and ICI 182,780 (Sigma-Aldrich, St Louis, MO, USA) were dissolved in absolute ethanol and the final concentration of the solvent in culture medium was 0.1% (v/v). 

 In the first experiment the response to OP was measured by comparing the testes from 18L : 6D and 6L : 18D males cultured in medium containing OP (at doses 10, 100, and 500 mg/L) with the testes cultured in medium containing solvent (control). In the second experiment (to examine the mechanism of OP action) testes were cultured in medium containing OP (at doses 10, 100, and 500 mg/L) or ICI (6 mg/L), or combination of each dose of OP plus 6 mg/L ICI. As a control, testes were exposed to medium containing solvent. 

 Doses of exposure were based on the literature [19]. Lower doses of OP (0.1, 1 mg/L) and ICI (2 mg/L) were tested in preliminary study. Since they appeared to have no effect on junction proteins, the data were not presented herein. After culture, the media and testes were collected and stored at −20°C and −80°C, respectively.

### 2.6. Immunohistochemistry

 To optimize immunohistochemical staining, slices were immersed for 4 min in 10 mM citrate buffer (pH 6.0) and heated in a microwave oven (600 W). The whole procedure has been described in detail elsewhere [20]. In short, nonspecific staining was blocked twice, first with 3% H_2_O_2_ in methanol for 15 min, to inhibit endogenous peroxidase activity, and second with 10% normal goat or horse serum (Sigma-Aldrich, St Louis, MO, USA) for 30 min at room temperature to block nonspecific binding sites. Thereafter, sections were incubated overnight at 4°C in a humidified chamber in the presence of primary antibodies: (1) a mouse monoclonal antibody against N-cadherin (1 : 100; Invitrogen, Eugene, OR, USA); (2) a rabbit polyclonal antibody against *β*-catenin (1 : 300; Invitrogen, Eugene, OR, USA); (3) a rabbit polyclonal antibody against Cx43 (1 : 2,000; Sigma-Aldrich, St Louis, MO, USA). Next, biotinylated secondary antibody, goat anti-rabbit IgG or horse anti-mouse IgG, respectively (1 : 400; Vector Labs., Burlingame, CA, USA) was applied for 1 h. Finally, avidin-biotinylated horseradish peroxidase complex (Vectastain ABC Kit; 1 : 100; Vector Labs., Burlingame, CA, USA) for a further 30 min was used. After each step in these procedures, sections were carefully rinsed with Tris-buffered saline (TBS; 0.05 M Tris-HCl plus 0.15 M NaCl, pH 7.6). Bound antibody was visualized with TBS containing 0.05% 3,3′-diaminobenzidine (Sigma-Aldrich, St Louis, MO, USA) and 0.07% imidazole (Sigma-Aldrich, St Louis, MO, USA) for 3-4 min. The slides were processed immunohistochemically at the same time with the same treatment so that the staining intensities among testicular cells could be compared. Experiments were repeated three times on serial sections per animal. Control sections included omission of the primary antibody. The sections were examined with a Leica DMR microscope (Leica Microsystems, Wetzlar, Germany).

### 2.7. Western Blot Analysis

 Tissues were homogenized on ice with a cold RIPA buffer (Sigma-Aldrich, St Louis, MO, USA), sonicated, and centrifuged at 15,000 g for 20 min at 4°C. The protein concentration for each sample was estimated using Bradford dye-binding procedure with bovine serum albumin (Sigma-Aldrich, St Louis, MO, USA) as a standard. Homogenates containing 100 *μ*g of protein were solubilized in a sample buffer (Bio-Rad Labs. GmbH, München, Germany) and boiled for 3 min. After denaturation the samples were subjected to electrophoresis on 10% polyacrylamide gels. Separated proteins were transferred onto a polyvinylidene difluoride (PVDF) membrane (Millipore Corporation, Billerica, MA, USA) using a wet blotter in the Genie Transfer Buffer (pH 8.4) for 90 min at a constant voltage of 135 V. Then blots were blocked with 5% nonfat dry milk in TBS, 0.1% Tween 20 for 1 h with shaking, followed by an incubation with appropriate primary antibody (as for immunohistochemistry; anti-N-cadherin, dilution 1 : 250; anti-*β*-catenin, dilution 1 : 800; anti-Cx43, dilution 1 : 10,000) overnight at 4°C. The membranes were washed and incubated with the horseradish-peroxidase labeled goat anti-rabbit IgG or horse anti-mouse IgG (Vector Labs., Burlingame, CA, USA) at a dilution 1 : 3,000, for 1 h at room temperature. Immunoreactive proteins were detected by chemiluminescence with Western Blotting Luminol Reagent (Santa Cruz Biotechnology, Santa Cruz, CA, USA), and images were captured with a ChemiDoc XRS+ System (Bio-Rad Labs. GmbH, München, Germany). All immunoblots were stripped with stripping buffer containing 62.5 mM Tris-HCL, 100 mM 2-mercaptoethanol and 2% SDS (wt/vol) (pH 6.7) at 50°C for 30 min, and incubated in a rabbit polyclonal antibody against *β*-actin (dilution, 1 : 3,000; Sigma-Aldrich) which served as a loading control. Each data point was normalized against its corresponding *β*-actin data point. Molecular masses were estimated by reference to standard proteins (Fermentas, GmbH, St. Leon-Rot, Germany). To obtain quantitative results, immunoblots were scanned using Image Lab 2.0 (Bio-Rad Labs. GmbH, München, Germany).

### 2.8. ELISA Assay

 Plasma FSH concentrations were measured with commercially available Mouse Follitropin subunit beta ELISA Kit (EIAab, Wuhan, China) according to the manufacturer's instructions. The sensitivity of the assay was 0.01 mIU/mL. The measurements were performed in duplicate. 

### 2.9. Statistical Analysis

 All statistical analyses were performed using one-way analysis of variance (ANOVA). Tukey multiple comparison test was used to determine which values differed significantly from controls (*P < 0.05, **P < 0.01, ***P < 0.001). Data were presented as mean ± SD.

## 3. Results

### 3.1. *In Vivo* Experiment

**Figure 1:**
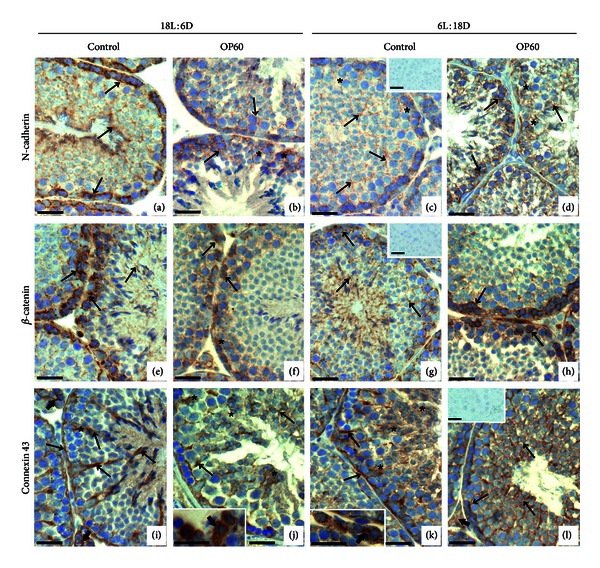
Immunohistochemical localization of N-cadherin (a)–(d), *β*-catenin (e)–(h), and connexin 43 (Cx43) (i)–(l) in the testes of bank voles kept in long (18L : 6D) or short (6L : 18D) photoperiod treated with vehicle (control) or 4-*tert*-octylphenol for 60 days (OP60). Scale bars represent 20 *μ*m. (a)–(d) Immunohistochemical localization of N-cadherin. (a) In control 18L : 6D males strong, linear staining at the region of blood-testis barrier and discrete punctuate staining in the adluminal compartment is observed (arrows). (b) Reduced intensity and delocalization of the staining following OP60 treatment (arrows). (c) In control 6L : 18D voles weak staining extends through much of the epithelium (arrows). (d) In OP60 tubules enhanced staining is visible (arrows). No signal was detected when anti-N-cadherin antibody was omitted (insert in (c)). (e)–(h) Immunohistochemical localization of *β*-catenin. (e) Typical distribution of *β*-catenin signal is seen in the basal compartment of seminiferous epithelium and at the regions of membrane apposition of adjacent Sertoli cell and elongated spermatids (arrows) in the testes of control 18L : 6D males. (f) Note decreased intensity of the staining at the blood-testis barrier site (arrows) and loss of the signal in the adluminal compartment in OP60 voles. (g) In the tubules of 6L : 18D animals weak staining is dispersed in the seminiferous epithelium (arrows). (h) Increased staining intensity in the basal compartment in OP60 males (arrows). No signal was detected when anti-*β*-catenin antibody was omitted (insert in (g)). (i)–(l) Immunohistochemical localization of connexin 43 (Cx43). (i) In the tubules of control 18L : 6D males Cx43 is seen predominantly between Sertoli cells and spermatogonia or pachytene spermatocytes and in the cytoplasm of some Sertoli cells as well as at Sertoli cell-spermatid junctions (arrows). Note strong linear staining between Leydig cells (small arrows). (j) In OP60 males Cx43 signal is localized in the form of irregular lines or distinct foci between the cells (arrows) and sometimes in the cytoplasm of Sertoli cells (asterisks). Cytoplasmic staining is present in most Leydig cells (small arrow; insert). (k) In the tubules of 6L : 18D voles irregular and discontinuous signal is visible (arrows). Frequently, the staining is confined to the cytoplasm of the cells (asterisks). In Leydig cells, staining of moderate intensity is detected in the cytoplasm (small arrow; insert). (l) Note altered staining pattern in OP60 animals; signal visible at the entire surfaces of Sertoli and germ cells (arrows). Very week staining in Leydig cell cytoplasm (small arrow). No signal was detected when anti-Cx43 antibody was omitted (insert in (l)).

 In control males expression and distribution of N-cadherin, *β*-catenin, and Cx43 were dependent on the length of the photoperiod. In seminiferous tubules of 18L : 6D animals strong, linear staining for N-cadherin and *β*-catenin was localized at the region of BTB, running parallel to the basement membrane. Additionally, discrete punctuate staining was observed in the adluminal compartment, mainly at Sertoli cell-elongated spermatid interface (Figures 1(a) and 1(e)). Cx43 was detected predominantly at the base of the tubules, between Sertoli cells and spermatogonia or pachytene spermatocytes as well as in the cytoplasm of some Sertoli cells. A moderate staining was also seen at Sertoli cell-spermatid junctions (Figure 1(i)). The localization and intensity of N-cadherin, *β*-catenin, and Cx43 immunostaining displayed some evidence of stage-specificity. In the Leydig cells, a very strong linear Cx43 signal on the plasma membrane was found as the prevalent staining pattern (Figure 1(i)).

 In tubules of 6L : 18D animals the intensity of N-cadherin and *β*-catenin staining in the basal region of the epithelium was clearly reduced when compared to 18L : 6D voles; weak, punctuate signal extended through much of the epithelium (Figures 1(c) and 1(g)). Similarly as in 18L : 6D animals, Cx43 was present predominantly in the basal compartment, however its distribution at the region of BTB was irregular and discontinuous. In the adluminal compartment weak staining was frequently dispersed in the cytoplasm of Sertoli or germ cells (Figure 1(k)). Cytoplasmic staining was also detected in Leydig cells of 6L : 18D males (insert in Figure 1(k)).

 Exposure to OP for 30 days did not alter the distribution of N-cadherin, *β*-catenin, and Cx43 neither in 18L : 6D nor 6L : 18D males (not shown). In males treated with OP for 60 days distribution pattern of these proteins was changed in tubules with altered spermatogenesis, as well as in some morphologically normal tubules. N-cadherin and Cx43 were frequently localized in the form of irregular lines or distinct foci between the cells (Figures 1(b) and 1(j)), in the cytoplasm of Sertoli cells (Figures 1(b), 1(d), and 1(j)), or at the entire surfaces of Sertoli and germ cells (Figure 1(l)). *β*-catenin reactivity remained at the region of BTB, whereas loss of the staining was detected in the apical compartment of seminiferous epithelium (Figures 1(f) and 1(h)). In 18L : 6D group signal intensities of all studied proteins were diminished following OP treatment when compared to the respective controls. In Leydig cells of OP60 males cytoplasmic staining for Cx43 was found (Figure 1(j)-insert, l). No staining was detected when the antibody against N-cadherin, *β*-catenin, or Cx43 was omitted (inserts in Figures 1(c), 1(g), and 1(l)).

**Figure 2:**
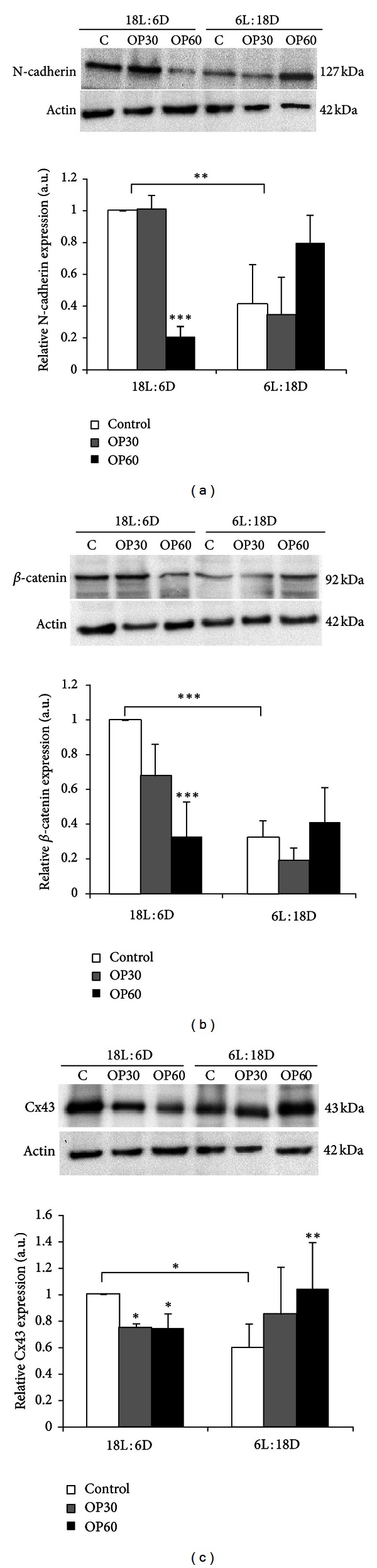
Representative Western blot analyses and relative N-cadherin (a), *β*-catenin (b), and connexin 43 (c) protein expression levels in testicular homogenates of bank voles kept in either long light cycles (18L : 6D) or short light cycles (6L : 18D) treated with vehicle (*n* = 6) or with 4-*tert*-octylphenol for 30 and 60 days (OP30, *n* = 6; OP60, *n* = 6). The relative amount of N-cadherin, *β*-catenin, and connexin 43 proteins normalized to *β*-actin. Data obtained from three separate analyses is expressed as mean ± SD. Significant differences from control values are denoted as *P < 0.05, **P < 0.01, and ***P < 0.001.

 Immunodetectable N-cadherin, *β*-catenin, and Cx43 proteins were observed as single bands near the 127, 92, and 43 kDa positions of the SDS gel, respectively, in testicular homogenates of the control voles and those treated with OP (Figure 2). In the testes of 18L : 6D males expression levels of N-cadherin and *β*-catenin were significantly decreased only in OP60 animals when compared to the respective controls (Figures 2(a) and 2(b)), whereas Cx43 was reduced in both OP30 and OP60 groups (Figure 2(c)). In 6L : 18D group an increase in the expression levels of all studied proteins was found. However, the increase was statistically significant only in case of Cx43 (Figure 2(c)). 

**Figure 3:**
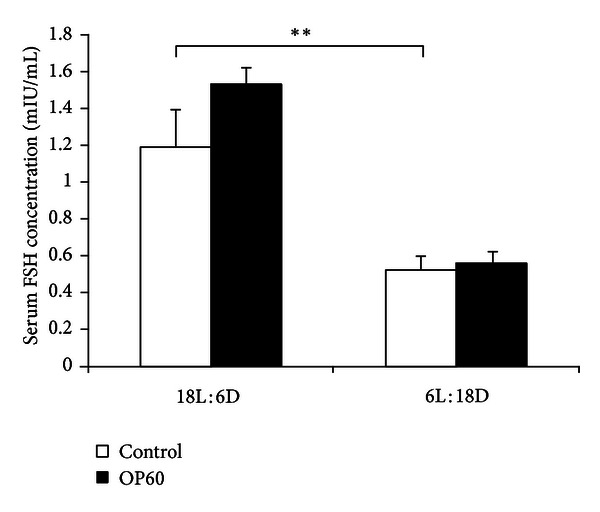
The effect of 4-*tert*-octylphenol treatment on FSH levels in the testes of 18L : 6D and 6L : 18D bank voles. Values are expressed as mean ± SD. Asterisks indicate significant differences between control animals (control, *n* = 6/each group) and males exposed to OP for 60 days (OP60, *n* = 6/each group). Statistical significance: **P < 0.01.

 Serum FSH concentration was higher in 18L : 6D animals when compared with 6L : 18D males. No significant influence of OP was seen on FSH concentrations in both 18L : 6D and 6L : 18D groups (Figure 3).

### 3.2. Organ Culture

**Figure 4:**
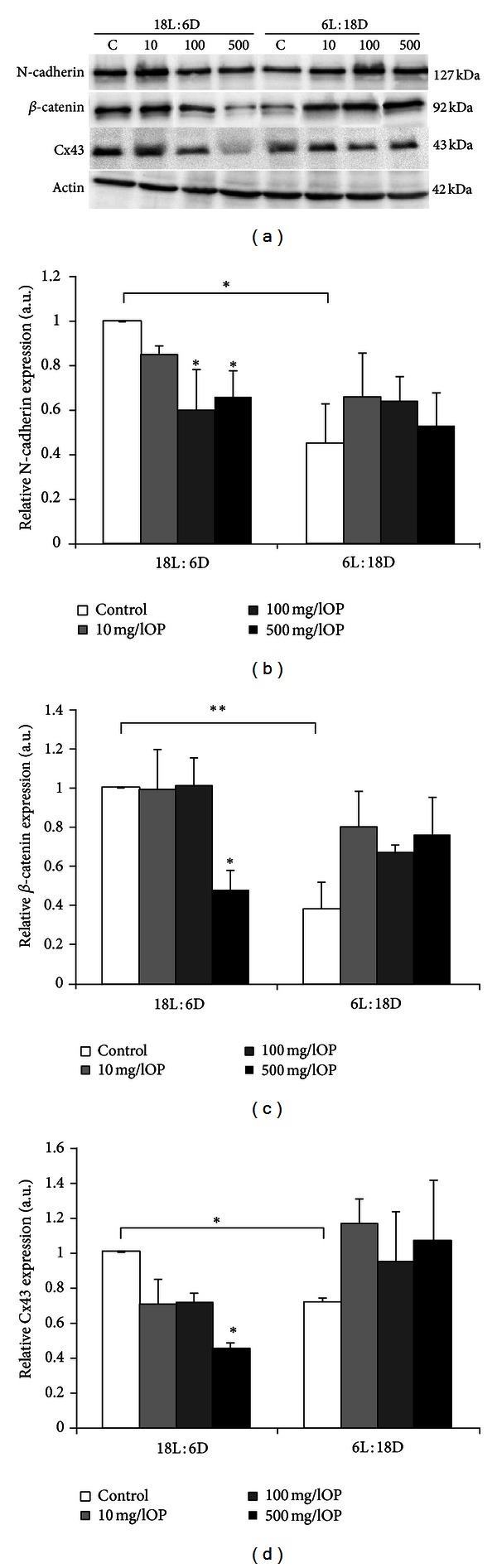
(a) Representative Western blot analysis of N-cadherin, *β*-catenin, and connexin 43 protein expression levels in homogenates of testis explants from bank voles kept in either long light cycles (18L : 6D; *n* = 8) or short light cycles (6L : 18D; *n* = 8). Explants were cultured in control medium (c) or in media containing 10, 100, or 500 mg/L 4-*tert*-octylphenol for 24 h. Anti-*β*-actin labeling served as an internal protein loading control. (b)–(d) The relative amount of N-cadherin (b), *β*-catenin (c), and connexin 43 (d) proteins normalized to *β*-actin. Data obtained from three separate analyses is expressed as mean ± SD. Significant differences from control values are denoted as *P < 0.05 and **P < 0.01.

When OP was present in the culture medium at concentrations of 10, 100, and 500 mg/L, in homogenates of testicular pieces from 18L : 6D animals a progressive decline in N-cadherin and Cx43 expression levels was obtained at all doses after 24 h of culture (Figures 4(b) and 4(d)); however only higher concentrations (100 and 500 mg/L) elicited statistically significant effect. Expression of *β*-catenin decreased following exposure to 500 mg/L OP, whereas lower doses had no effect. In testis explants of 6L : 18D males protein expression levels were increased, but a relatively large variation in the expressions of N-cadherin and *β*-catenin between the individual testes exposed to OP meant the differences were not statistically significant (Figures 4(b)–4(d)).

**Figure 5:**
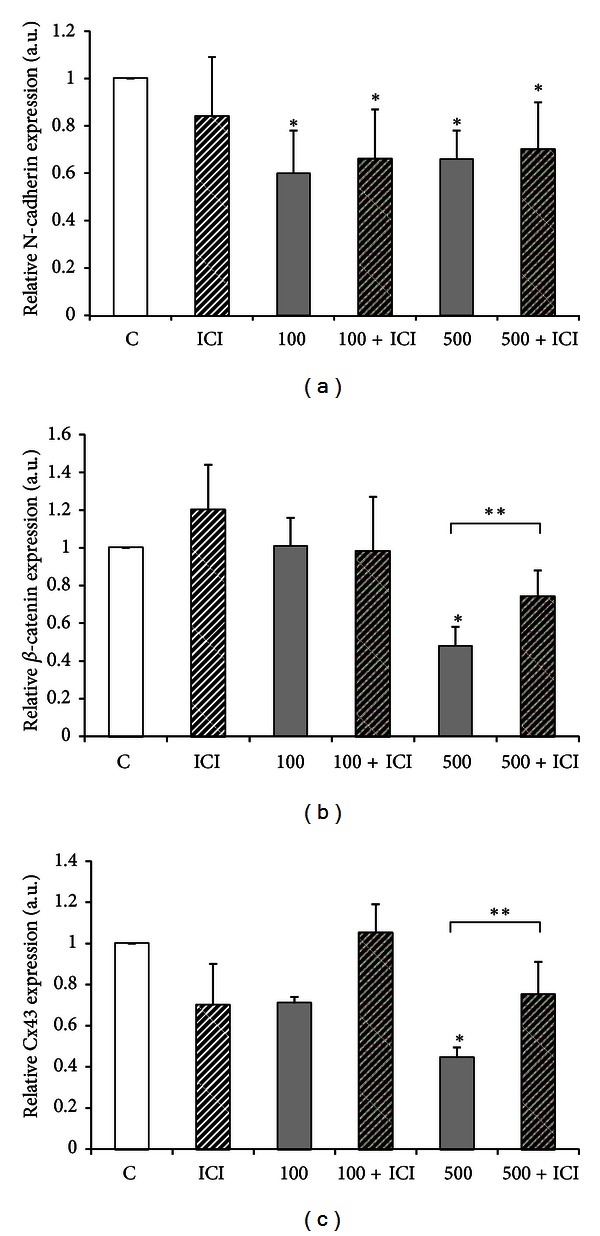
The effects of ICI 182,780 on octylphenol-induced decrease of N-cadherin (a), *β*-catenin (b) and connexin 43 (c) protein expression in homogenates of testis explants from bank voles kept in long light cycles (*n* = 8). Explants were cultured in control medium (c) or in media containing 6 mg/L ICI 182,780 (ICI); 100 or 500 mg/mL 4-*tert*-octylphenol (100, 500); 100 or 500 mg/mL 4-*tert*-octylphenol and 6 mg/L ICI 182,780 (100+ICI; 500+ICI) for 24 h. Graphs represent the relative amount of N-cadherin (b), *β*-catenin (c), and connexin 43 (d) protein normalized to *β*-actin. Data obtained from three separate analyses is expressed as mean ± SD. Significant differences from control values are denoted as *P < 0.05 and **P < 0.01.

 An inhibitory experiment was performed using pure antiestrogen, ICI 182,780. Explants from 18L : 6D males were tested with 6 mg/L ICI alone for 24 h; 100 and 500 mg/L OP alone for 24 h; or combination of each dose of OP plus 6 mg/L ICI. Since ICI showed no discernible effect on proteins' expression, solvent-treated explants served as controls in this experiment. Coadministration of ICI did not block the effects of OP on N-cadherin expression (Figure 5(a)). On the other hand, ICI partially reversed the OP-induced decrease in *β*-catenin and Cx43 in testicular fragments cultured *in vitro *(Figures 5(b) and 5(c)). 

## 4. Discussion

 To assess the effects of OP on junction proteins expression in bank vole testis* in vivo* and *in vitro* methods were used. *In vivo* experiments are required for understanding the action of tested chemical in the internal environment of the organism, where it undergoes various biochemical transformations, affecting their bioavailability and activity. It was reported that in mammals OP is metabolized by the liver to two glucuronide conjugates [21]. Glucuronidation of OP eliminates its estrogen-like activity; however glucuronides may be hydrolysed back to active compounds [22]. At doses that exceed the metabolization level, OP accumulates in various organs, particularly, in adipose tissue, liver, muscles, and brain [23, 24]. Therefore, the effects of OP action are noted following administration of high doses or after chronic exposure, as it was demonstrated by data obtained in our and other laboratories [17, 25]. In addition, the influence of OP on endogenous estrogens production might contribute to the effects observed *in vivo *[17].

In contrast, during *in vitro* organ culture of the testis the influence of extratesticular factors is avoided, while relationship among the cells remains intact and the interactions between the cells can be accurately evaluated [26]. Therefore, short-term organ culture was used to examine whether OP could exert direct and immediate effects on junction proteins expression in the cells of the seminiferous tubule.

The results presented herein demonstrate that exposure of male bank voles to OP has a potential to induce adverse effects on junction proteins in the testes. Interestingly, alterations in the expressions and localizations of these proteins appeared to be dependent on the length of the photoperiod. Our results are the first reported on the influence of the length of the photoperiod on N-cadherin and *β*-catenin proteins in the testis of seasonally breeding rodents. It should be noted that bank voles kept under long photoperiod (18L : 6D) show similar reproductive characteristics as those observed in the wild in reproductively active males during spring and summer, whereas animals exposed to short light regime (6L : 18D) show regressive phase of reproduction that occurs in the wild voles during late autumn and winter. In the testes of males kept in short light cycles most of seminiferous tubules exhibit small or no lumen with not fully differentiated germ cells. Decrease in the level of plasma gonadotropins during the short photoperiod results in reduced testosterone production decreased expression and activity of steroidogenic enzymes and reduced expression of androgen receptor when compared to long-day animals [17, 27, 28]. The present study provides clear evidence that in bank voles also direct cell-cell interactions are regulated by length of the light cycle, since the expression and localization of protein markers of adherens and gap junctions are different in animals reared under different light conditions. We noticed that organization of N-cadherin/*β*-catenin and Cx43-based junctions in the seminiferous epithelium of 18L : 6D males was consistent with the organization of morphologically mature junctions in the adult testes of continual breeders such as mice and rats [11, 13, 29, 30]. On the contrary, in 6L : 18D animals distribution of junction proteins was similar to those observed in immature or prepubertal males, in which weaker, punctuate, or cytoplasmic staining was dispersed in the basal and adluminal compartment of the epithelium [13, 31]. This is in line with the results of Tarulli et al. [32], who reported that in short-day males of seasonally breeding Djungarian hamster Sertoli cells have characteristics of both adult and immature phenotypes, suggesting that they take on an intermediate or transitional state. It should be mentioned that early study by Pelletier [33] demonstrated the localization of Cx43 in another seasonal breeder, mink. In contrast to our observations, the author reported that during winter testicular regression immunoexpression of Cx43 is present only in the basal third of the epithelium. Such a result was presumably a consequence of performing less sensitive immunohistochemical method with the use of secondary antibody conjugated directly to peroxidase.

 Disorganization and decreased expressions of N-cadherin, *β*-catenin, and Cx43 proteins in 6L : 18D bank vole males were accompanied with reduced FSH level in these animals. 

There is evidence that FSH stimulates the formation of extensive inter-Sertoli cell adherens junctions; in the absence of FSH adherens junction puncta were observed in rat Sertoli cells *in vitro*, whereas the addition of FSH induced the reorganization of these puncta into adherens junction belts [34]. Also gap junction coupling and organization of Cx43 gap junction plaques between Sertoli cells *in vitro *appeared to be regulated by FSH and cAMP [35, 36]. Moreover, recent data on Djungarian hamsters indicate that gonadotropin suppression induced by short photoperiod disrupted distribution and expressions of tight junction proteins, and FSH replacement led to a rapid reorganization of these proteins [32]. Based on these data and our results we believe that in bank voles FSH could be a factor controlling adherens and gap junctions arrangement in the seminiferous epithelium during transition from reproductive quiescence to reproductive activity.

 Despite numerous studies on testis histopathology of males treated with OP, very little is known about the alterations induced by this xenoestrogen on cell–cell junction molecules. In the only paper reporting the effects of OP on Sertoli cell junction proteins, Fiorini et al. [37] observed reduction in the levels of occludin, N-cadherin, and Cx43 in the SerW3 Sertoli cell line treated with 0.2 *μ*M OP for 24 h. In addition to its effect on protein levels, OP was able to delocalize the proteins from the membrane to the cytoplasmic compartment [37]. In the present study, it was shown that long-term *in vivo* OP treatment of adult bank voles also resulted in the reduction of N-cadherin, *β*-catenin, and Cx43 proteins expressions and their delocalization, but only in the testes of males kept in long photoperiod. Surprisingly, in short-day animals slight increase of Cx43 (*P* < 0.05), N-cadherin, and *β*-catenin (statistically nonsignificant) levels was found. These differences appeared to be independent of FSH, since OP treatment did not change FSH concentrations neither in long-day nor in short-day animals. Thus, we hypothesize that OP affects junction proteins expression acting directly on the testis. To test this hypothesis we used organ culture model. We found that in testis explants cultured with OP for 24 h expression levels of all studied proteins were reduced in long-day voles and slightly elevated in short-day animals. Photoperiod-dependent effect of OP on junction proteins in bank vole testes was therefore maintained in *in vitro* conditions, indicating that hypothalamic-pituitary axis is not involved in this effect. 

 The reason for diverse responses of males kept in different light conditions to OP remains to be elucidated. Nevertheless, since in our previous studies on bank vole testis we found elevated estradiol concentrations and aromatase expression following OP exposure [17], it is possible that in short-day voles OP directly acting (as estrogen-like compound), or more likely through induction of local estradiol production, restores the expression levels of junction proteins. These hypothesis is based on our earlier observations that exposure to low dose of exogenous estradiol induced acceleration of the onset of spermatogenesis in voles kept under short light cycle conditions and on the finding that estrogens have FSH-independent stimulatory effect on spermatogenesis in photoregressed Siberian hamster [38, 39]. 

 On the other hand, in long-day males, OP exerts negative effect on the expression of junction proteins, presumably by inducing supraphysiological estrogen level or action. Indeed, our previous studies revealed that in bank vole males treated with a high dose of estradiol disruption of testicular structure and tubular atrophy occurred [38]. Moreover, in rats exogenous estrogens appeared to affect cell-cell junctions in the testis; 17*β*-estradiol was shown to be disruptive to the BTB integrity, while 17*α*-ethinylestradiol altered intercellular communication by disrupting gap junction functionality and Cx43 trafficking in cultured Sertoli cells as well as in isolated rat seminiferous tubules [12, 40]. 

 It should be noted that in our study OP had the most rapid effects on Cx43 protein expression in the testes of 18L : 6D males (visible just after 30-day exposure). This observation argues for the recent hypothesis that Cx43 is one of the main targets for endocrine disruptors and that impaired Cx43 expression affects other proteins of the BTB [41]. 

 The question arises as to the mechanism of OP action on junction proteins in the seminiferous tubules. To determine whether this actions was mediated through binding to ER*α* or ER*β*, testicular explants from 18L : 6D males were treated concomitantly with a pure estrogen antagonist, ICI 187 780 (6 mg/L), and increasing OP concentrations (10 to 500 mg/L). We found that *β*-catenin and Cx43 OP-induced decrease was partially blocked by ICI, suggesting that the biological effects of OP on the expression of these proteins involve an ER-mediated response. On the other hand, the influence of OP on N-cadherin does not appear to be mediated through the classic nuclear ERs, since ICI did not alter the effects of OP. Several earlier papers reported that also some other effects of OP were not mediated through the ER, possibly involving interactions with membrane components or receptors. For example, Murono et al. [42, 43] described the effects of OP on the conversion of progesterone to testosterone and hCG-stimulated testosterone biosynthesis as independent of ER*α* and ER*β*. 

It cannot be, however, excluded that after the relatively short incubation period (24 h) some amounts of the proteins are not degraded and still exist in the cultured tissue, even if the mRNA level was downregulated. Therefore, further studies including sequencing of bank vole genes and the application of RT-PCR method are needed to verify data regarding mechanisms of OP action on junction proteins expression in the bank vole testes. 

## 5. Conclusion

 In this study we demonstrated that in seasonally breeding bank vole organization of adherens and gap junctions as well as the expression of junction proteins is related to the length of photoperiod and, in consequence, to the reproductive status of the animal. In addition, we found that reproductive status may determine response to OP. Finally, we found that alterations in cadherin/catenin and Cx43-based junction in the testes following OP exposure may partially result from activation of ER*α* and/or ER*β* signaling pathway.
